# Identification of circRNA circ-CSPP1 as a potent driver of colorectal cancer by directly targeting the miR-431/LASP1 axis

**DOI:** 10.1515/biol-2021-0053

**Published:** 2021-05-29

**Authors:** Minghao Li, Jianbin Zhuang, Di Kang, Yuzhuo Chen, Weiliang Song

**Affiliations:** Department of Gastrointestinal Anorectal Surgery, Tianjin Third Central Hospital, No. 83, Jintang Road, Hedong District, Tianjin, 300170, China

**Keywords:** colorectal cancer, circ-CSPP1, miR-431, LASP1

## Abstract

Colorectal cancer (CRC) is the third most common malignancy worldwide. Circular RNAs (circRNAs) have been implicated in cancer biology. The purpose of the current work is to investigate the precise parts of circRNA centrosome and spindle pole-associated protein 1 (circ-CSPP1) in the progression of CRC. Our data showed that circ-CSPP1 was significantly overexpressed in CRC tissues and cells. The knockdown of circ-CSPP1 attenuated cell proliferation, migration, invasion and promoted apoptosis *in vitro* and weakened tumor growth *in vivo*. circ-CSPP1 directly targeted miR-431, and circ-CSPP1 knockdown modulated CRC cell progression *in vitro* via upregulating miR-431. Moreover, LIM and SH3 protein 1 (LASP1) was a functional target of miR-431 in modulating CRC cell malignant progression. Furthermore, circ-CSPP1 in CRC cells functioned as a posttranscriptional regulator on LASP1 expression by targeting miR-431. Our present study identified the oncogenic role of circ-CSPP1 in CRC partially by the modulation of the miR-431/LASP1 axis, providing evidence for circ-CSPP1 as a promising biomarker for CRC management.

## 1 Introduction

Colorectal cancer (CRC) is the third most prevalent malignancy worldwide as of 2018, accounting for approximately one in ten cancer cases and deaths [1]. Although new treatment options have markedly increased the overall survival of non-metastasized CRC, the prognosis remains very poor for those with advanced disease [2]. Currently, biomarkers are considered as important tools in early detection and clinical therapy of patients with CRC [3]. Thus, there is an urgent need to identify novel and effective biomarkers for managing CRC.

Circular RNAs (circRNAs), mostly formed by head-to-tail splicing of exons, are an important family of posttranscriptional regulators in eukaryotes [4]. circRNAs are attracting considerable attention due to their microRNA (miRNA) regulation in almost all cancers, including CRC [5,6]. For instance, Yuan et al. highlighted hsa_circ_0026344 as a novel prognostic biomarker for CRC patients by regulating miR-21 and miR-31 [7]. Li et al. identified hsa_circ_0006990 as a negative regulator of miR-101, with the ability to accelerate CRC tumorigenesis [8]. Bian et al. illuminated that hsa_circ_103809 controlled the carcinogenesis of CRC through targeting miR-532-3p [9]. As for circRNA centrosome and spindle pole-associated protein 1 (circ-CSPP1, hsa_circ_0001806), an upregulated circRNA in CRC, it was reported to be implicated in the stemness of CRC cells through the modulation of miR-193-5p [10]. However, no insight has been gained into the precise parts of circ-CSPP1 in CRC progression.

miRNAs, an evolutionarily conserved class of small RNAs, are able to silence gene expression during tumorigenesis [11]. Abnormal miRNA expression has been demonstrated in CRC [12,13]. Su and Liu uncovered that miR-431 (also called miR-431-5p) was underexpressed in CRC and functioned as a potent antitumor miRNA in this disease by directly targeting cullin-4B [14]. Nevertheless, whether miR-431 is a downstream mediator of circ-CSPP1 in controlling CRC development has not been described.

The gene expression regulation of circRNAs exhibited by miRNAs [15] led us to hypothesize that circRNA–miRNA–mRNA networks might serve as crucial modulators of CRC development. Here, we identified a significantly overexpressed circRNA, circ-CSPP1, participated in CRC development through targeting the miR-431/LIM and SH3 protein 1 (LASP1) axis.

## 2 Materials and methods

### 2.1 Clinical specimens and human study

Primary CRC tissues and paired non-tumor tissues from 37 patient volunteers were collected from the Tianjin Third Central Hospital. The clinicopathological data of these patients are provided in Table 1. A portion of the specimens was stored at −80°C and used to investigate circ-CSPP1, miR-431, and LASP1 mRNA expression levels and analyze their correlations. Also, a portion of the specimens was used for histological analysis by two pathologists using hematoxylin and eosin (H&E) staining. Briefly, CRC samples were fixed in formaldehyde (Solarbio, Beijing, China), blocked in paraffin, and then sectioned at 4 µm intervals. The sections were stained with hematoxylin (Solarbio) for 5 min and eosin (Solarbio) for 3 min, followed by the observation of the images under a microscope (Leica, Wetzlar, Germany).

**Table 1:** Correlation between circ-CSPP1 expression and the clinicopathological features of CRC patients

	Characteristics (*n* = 37)	circ-CSPP1 expression		*P* value
		Low (*n* = 18)	High (*n* = 19)	
**Gender**				0.6185
Female	19	10	9	
Male	18	8	10	
**Age (years)**				0.6378
≤60	15	8	7	
>60	22	10	12	
**TNM grade**				0.0138*
I + II	17	12	5	
III + IV	20	6	14	
**Lymph node metastasis**				0.0051*
Positive	21	6	15	
Negative	16	12	4	
**Tumor size (cm)**				0.0005*
≤3	16	13	3	
>3	21	5	16	

*P < 0.05 by Chi-square test.


**Informed consent:** Informed consent has been obtained from all the individuals included in this study.
**Ethical approval:** The research related to human use has been complied with all the relevant national regulations, institutional policies and in accordance with the tenets of the Helsinki Declaration; and the research has been approved by the Ethics Committee of Tianjin Third Central Hospital.

### 2.2 Cell culture and transfection

Four CRC cell lines SW620, SW480, LOVO, and HCT-116 (American Type Culture Collection, Manassas, VA, USA) were grown in Dulbecco’s Modified Eagle’s Medium (DMEM; Thermo Fisher Scientific, Paisley, UK) with 10% fetal calf serum (EuroClone, Milan, Italy) at 37°C and 5% CO_2_. Noncancerous NCM460 colon cells (INCELL, San Antonio, TX, USA) were propagated using M3 Base Medium provided by INCELL.

The plasmid construct overexpressing circ-CSPP1 was generated by cloning the overall length of hsa_circ_0001806 into a pCD25-ciR vector (Geneseed, Guangzhou, China), and the control plasmid (vector) was produced by using a scrambled sequence. LASP1 overexpression plasmid was created by introducing human LASP1 (NM_006148.4) into the pcDNA3.1 vector (Invitrogen, Merelbeke, Belgium) with BamH I and Xho I restriction sites. These constructs were transiently transfected into HCT-116 and SW480 cells (∼50% confluence) at 100 ng with Lipofectamine 2000 (Thermo Fisher Scientific). Lentiviruses encoding circ-CSPP1-shRNA (sh-circ-CSPP1) or nontarget-shRNA (sh-NC) were obtained from Geneseed and used as per the manufacturing instructions. circ-CSPP1 was silenced in HCT-116 and SW480 cells by transiently transfecting circ-CSPP1-siRNA (50 nM, si-circ-CSPP1, 5′-AUUUGGAUGUUUCAUUGUCUG-3′; GenePharma, Shanghai, China) or in SW480 cells by stably transducing cells with sh-circ-CSPP1. Also, miR-431 overexpression and knockdown in HCT-116 and SW480 cells were achieved by using miR-431 mimic (5′-UGUCUUGCAGGCCGUCAUGCA-3′) and matched inhibitor (anti-miR-431, 5′-UGCAUGACGGCCUGCAAGACA-3′), respectively. Negative oligonucleotide controls were nontarget-siRNA (si-NC, 5′-AAGACAUUGUGUGUCCGCCTT-3′), miR-NC mimic (5′-ACGUGACACGUUCGGAGAATT-3′), and anti-miR-NC (5′-CAGUACUUUUGUGUAGUACAA-3′, all from GenePharma). The transfected cells were analyzed 48 h after transfection for gene expression and functional investigation.

### 2.3 RNA extraction and ribonuclease R (RNase R) assay

Total RNA extracted from clinical specimens and cells with the RNeasy Mini Kit (Cat. 74106; Qiagen, Tokyo, Japan) was adjusted to a concentration of 10 ng/µL when quantified with a NanoDrop (Thermo Fisher Scientific). Total RNA (1 µg) from HCT-116 and SW480 cells was digested with 3 U of RNase R (Geneseed) for 15 min at 37°C, followed by the purification with the RNeasy MinElute Cleanup Kit (Cat. 74204; Qiagen) as per the guidance of manufacturers.

### 2.4 Subcellular fractionation

Nuclear RNA and cytoplasmic RNA were isolated from HCT-116 and SW480 cells with the Cytoplasmic & Nuclear RNA Purification Kit (Cat. NGB-37400; Norgen Biotek, Thorold, ON, Canada) as recommended by the manufacturers. RNU6 (U6) and glyceraldehyde-3-phosphate dehydrogenase (GAPDH) were used as the controls.

### 2.5 Quantitative reverse transcription polymerase chain reaction (qRT-PCR)

To measure circ-CSPP1 and mRNA levels, cDNA preparation was done in 25 µL reactions using ReverTra Ace (Cat. 410800; Toyobo, Tokyo, Japan) with 1 µg total RNA and 250 ng random primers (Invitrogen); qRT-PCR analysis using SYBR qPCR Mix (Cat. QPS-201; Toyobo) plus 25 ng of both the forward and reverse primers (Table 1) was run on a LightCycler DX400 (Roche, Mannheim, Germany). To quantify miR-431 expression, the MultiScrible Reverse Transcriptase (Cat. 4311235; Invitrogen) for cDNA preparation and TaqMan Universal PCR Master Mix (Cat. 4364340; Applied Biosystems, Paisley, UK) with designed primers (Table A1) for qRT-PCR were used as per the accompanying protocols. Using the comparative Ct method (2^−ΔΔCt^ with logarithm transformation) [16], the results were normalized on the basis of control GAPDH or U6 expression.

### 2.6 Cell viability and colony formation assays

For cell viability assay, transiently transfected HCT-116 and SW480 cells in 96-well plates at an initial density of 1 × 10^3^ cells per well were cultured in DMEM growth media for 48 h, and the media was replaced with medium containing 10 µL per well of the Cell Counting Kit-8 (CCK-8) substrate (Cat. ab228554; Abcam, Cambridge, UK). Following the 37°C incubation for 2 h, Varioskan Flash (Thermo Fisher Scientific) was used for the measurement of the absorbance at 460 nm. For colony formation assay, transiently transfected HCT-116 and SW480 cells in 6-well plates at 150 cells per well were cultured in DMEM growth media. Colonies were allowed to grow for 14–21 days before imaging and counting. The number of colonies (over 50 cells) was manually counted to get an average of three fields after being stained with 0.5% crystal violet (Solarbio).

### 2.7 Flow cytometry

A total of 1 × 10^6^ transiently transfected HCT-116 and SW480 cells were incubated with Annexin V-fluorescein isothiocyanate (BD Biosciences, North Ryde, NSW, Australia) and propidium iodide (PI; Invitrogen) for 15 min in the dark and analyzed within 1 h with a flow cytometer (BD Biosciences).

### 2.8 Transwell migration and invasion assays

Transiently transfected HCT-116 and SW480 cells were seeded onto the non-coated membrane (24-transwell insert, 8 µm; Corning, Rochester, NY, USA) at 2.5 × 10^4^ cells per well for migration assay and plated onto the Matrigel-precoated membrane (BD Biosciences) in the insert of a 24-well plate at 5 × 10^4^ for invasion assay. Media plus 10% fetal calf serum was used as a chemoattractant in the lower chamber. After the 37°C incubation for 24 h, the migratory or invasive cells were stained with 0.5% crystal violet and counted under a 100× magnification microscope.

### 2.9 Western blot

Protein preparation from clinical specimens and transfected or untransfected cells was conducted as reported in ref. [17]. For immunoblotting, total protein (50 µg) was resolved on denaturing gels with 4–12% SDS-polyacrylamide and transferred to polyvinylidene difluoride membranes (BD Biosciences) by wet transfer. The membranes were probed with primary antibodies, including anti-Ki-67 (ab92742; dilution 1:5,000), anti-B cell lymphoma 2 (anti-Bcl-2, ab117115; dilution 1:1,000), anti-matrix metallopeptidase 9 (anti-MMP9, ab76003; dilution 1:3,000), anti-LASP1 (ab117806; dilution 1:5,000), and anti-GAPDH (ab186930; dilution 1:1,000), and IgG secondary antibodies labeled by horseradish peroxidase (ab205719 and ab97051, all from Abcam; dilution 1:10,000 and 1:10,000). Signal detection was done by the Enhanced Chemiluminescence (Cat. 34578; Thermo Fisher Scientific) before analyzing the band densitometry.

### 2.10 Bioinformatics and dual-luciferase reporter assay

The targeted miRNAs of circ-CSPP1 were predicted by online CircInteractome database. miRNA-binding sites were searched by online software starBase v.3. The genomic fragments of circ-CSPP1 and LASP1 3′-UTR encompassing the miR-431-binding region or mutated seed sequence were individually ligated into the psiCHECK-2 vector (Promega, Southampton, UK). These reporters (100 ng) were cotransfected with 25 nM of miR-431 mimic or control mimic into either HCT-116 or SW480 cells (1 × 10^5^) in 24-well plates. Post 48 h of incubation, luciferase assays were done using the Dual-luciferase Assay System (Promega).

### 2.11 Xenograft model studies

For the establishment of the xenograft model, 6- to 8-week-old BALB/c female mice (*n* = 12; Vital River Laboratory, Beijing, China) were used. Also, 2 × 10^6^ sh-NC- or sh-circ-CSPP1-transduced SW480 cells in 200 µL of 50% Matrigel Matrix (BD Biosciences) were subcutaneously inoculated into the flanks of BALB/c mice (*n* = 6 per group). Tumor volume was periodically measured with a digital caliper and estimated using the (length × width^2^) × 0.5 formula. At end points, mice were euthanized to harvest the xenograft tumors.


**Ethical approval:** The research related to animal use has been complied with all the relevant national regulations and institutional policies for the care and use of animals; and the research has been approved by the Animal Care and Use Committee of Tianjin Third Central Hospital.

### 2.12 Immunohistochemistry and terminal deoxynucleotidyl transferase dUTP nick-end labeling (TUNEL) assay

The xenograft tumors were fixed with 4% neutral-buffered formalin, dehydrated, embedded in paraffin, and sectioned at 5 µm intervals. Proliferation and invasion of the xenograft tumors were evaluated by immunohistochemistry using anti-Ki-67 (ab21700; Abcam; dilution 1:1) and anti-MMP9 (ab228403; Abcam; dilution 1:5,000) antibodies, respectively. Briefly, paraffin-embedded sections were dewaxed, heated in 0.01 M of sodium citrate buffer, and treated with methanolic H_2_O_2_, followed by the incubation with anti-Ki-67 and anti-MMP9. Immunoreactive signals were detected using 3,3′-diaminobenzidine substrate (Thermo Fisher Scientific), and then the sections were counterstained with hematoxylin and mounted. Apoptosis of the tumor cells was assessed by staining paraffin-embedded sections with the TUNEL Assay Kit (Cat. ab206386; Abcam) as per the accompanying guidance.

### 2.13 Statistical analysis

All experiments were carried out in triplicate, and the results were presented as mean ± standard deviation. The Mann–Whitney *U* test, Student’s *t*-test, and analysis of variance were used to compare the measured parameters. The correlations between miR-431 and circ-CSPP1 or LASP1 expression in cancer tissues were judged by a statistic test based on Pearson’s correlation coefficient. For survival data, the Kaplan–Meier method and log-rank tests were used. The values were statistically significant at *P < 0.05, **P < 0.01, ***P < 0.001, or ****P < 0.0001.

## 3 Results

### 3.1 circ-CSPP1 was overexpressed in CRC tissues and cells

**Figure 1:**
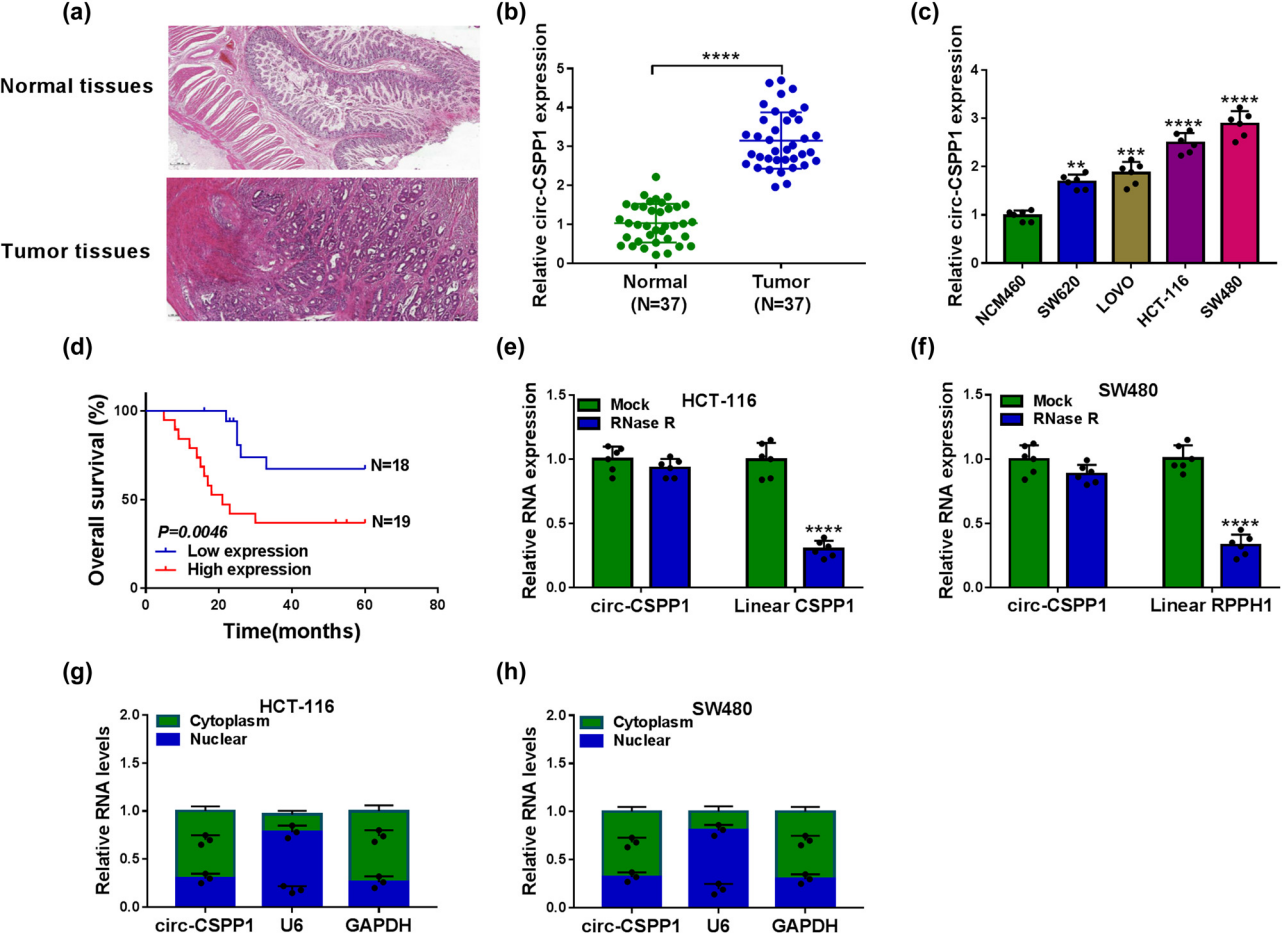
The level of circ-CSPP1 was upregulated in CRC tissues and cells. (a) H&E staining of cancer tissues and paired noncancerous tissues. (b) The relative level of circ-CSPP1 by qRT-PCR in 37 pairs of cancer tissues and matched normal tissues. (c) Relative circ-CSPP1 expression by qRT-PCR in NCM460, SW620, SW480, LOVO, and HCT-116 cells. (d) Overall survival analyses based on the level of circ-CSPP1 in cancer tissues of these CRC patients divided according to the median of circ-CSPP1 level. (e and f) RNase R digestion assay in HCT-116 and SW480 cells. (g and h) Subcellular localization assay in HCT-116 and SW480 cells. **P < 0.01, ***P < 0.001, or ****P < 0.0001.

To observe the involvement of circ-CSPP1 in CRC development, we used qRT-PCR to determine its expression in cancer tissues and paired noncancerous tissues, which were identified by H&E staining (Figure 1a). Remarkably, circ-CSPP1 was upregulated in CRC tissues compared with normal tissues (Figure 1b). Similarly, by contrast, circ-CSPP1 level was strikingly augmented in CRC cells (Figure 1c). To evaluate the clinical significance of circ-CSPP1 in CRC, we analyzed the correlation between the overall survival of patients and circ-CSPP1 level. As expected, Kaplan–Meier survival curves revealed that the patients with low circ-CSPP1 expression had a longer survival time than those with high circ-CSPP1 level (Figure 1d). Furthermore, the expression of circ-CSPP1 was closely associated with the tumor node metastasis (TNM) grade, lymph node metastasis, and size of these tumors (Table 1). Moreover, the digestion with RNase R led to a striking reduction in the level of corresponding linear mRNA (Linear CSPP1), and circ-CSPP1 was resistant to RNase R (Figures 1e and f), demonstrating the stability of circ-CSPP1. Additionally, subcellular localization results revealed that circ-CSPP1 was mainly localized in the cytoplasm of HCT-116 and SW480 cells (Figure 1g and h).

### 3.2 Knockdown of circ-CSPP1 attenuated CRC cell proliferation, migration, invasion and enhanced apoptosis *in vitro*

**Figure 2:**
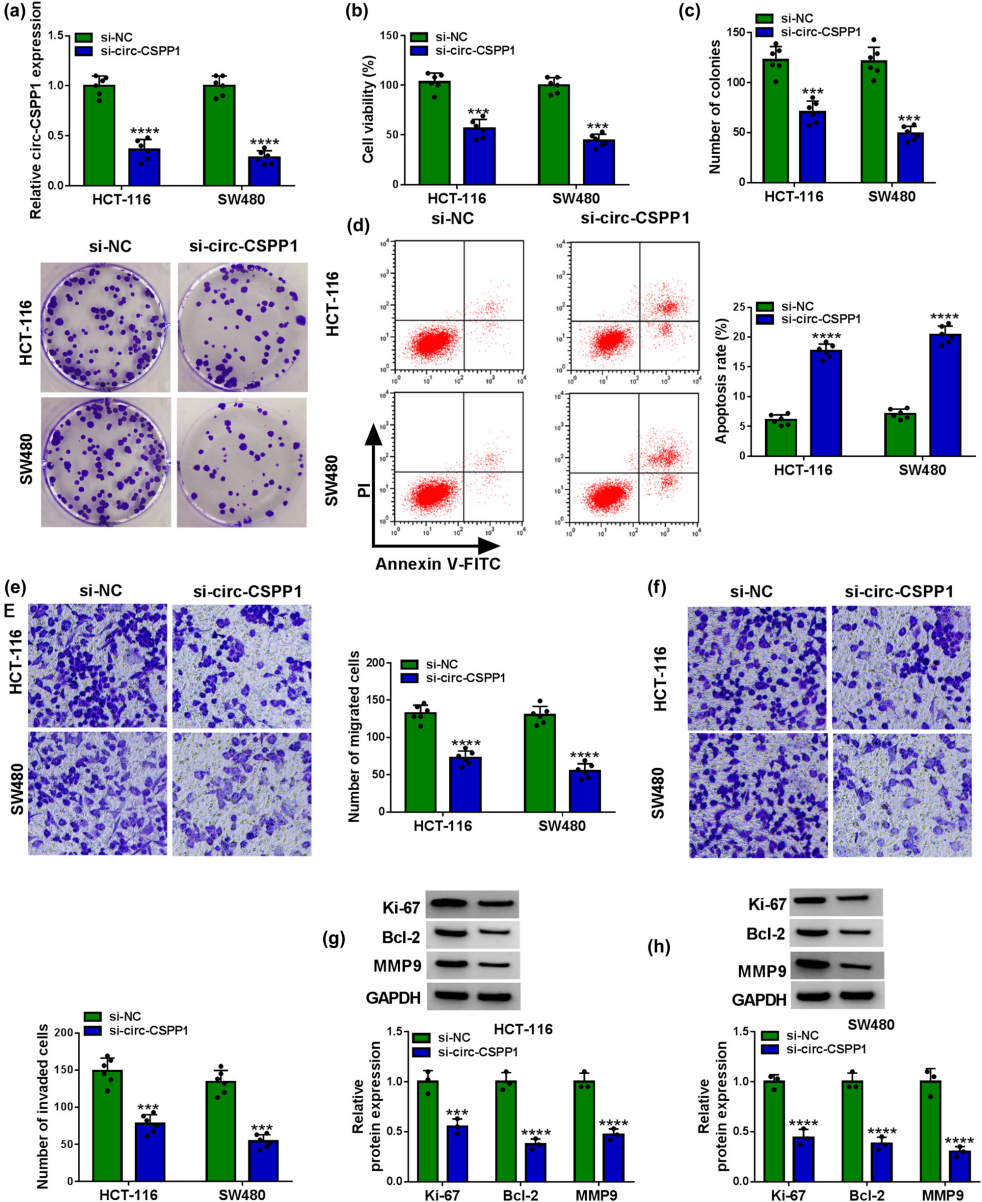
circ-CSPP1 knockdown attenuated CRC cell progression *in vitro* by regulating cell proliferation, migration, invasion, and apoptosis. HCT-116 and SW480 cells were transfected with si-NC or si-circ-CSPP1, followed by the assessment of relative circ-CSPP1 expression by qRT-PCR (a), cell viability by CCK-8 assay (b), cell colony formation by colony formation assay (c), cell apoptosis by flow cytometry (d), cell migration and invasion by transwell assay (e and f), the relative protein levels of Ki-67, Bcl-2, and MMP9 by western blot (g and h). ***P < 0.001 or ****P < 0.0001.

We then carried out loss-of-function analyses *in vitro* using circ-CSPP1-siRNA (si-circ-CSPP1). Transient transfection of si-circ-CSPP1, but not the si-NC control, caused a remarkable downregulation of circ-CSPP1 level in both HCT-116 and SW480 cells (Figure 2a). In contrast, the knockdown of circ-CSPP1 led to a striking inhibition in cell viability (Figure 2b) and colony formation (Figure 2c), and a significant promotion in cell apoptosis (Figure 2d), as well as a prominent repression in cell migration (Figure 2e) and invasion (Figure 2f). Furthermore, western blot results showed that circ-CSPP1 knockdown resulted in reduced levels of proliferation marker Ki-67, anti-apoptotic protein Bcl-2, and metastasis-related protein MMP9 in the two CRC cells (Figure 2g and h).

### 3.3 circ-CSPP1 directly targeted miR-431 in CRC cells

**Figure 3:**
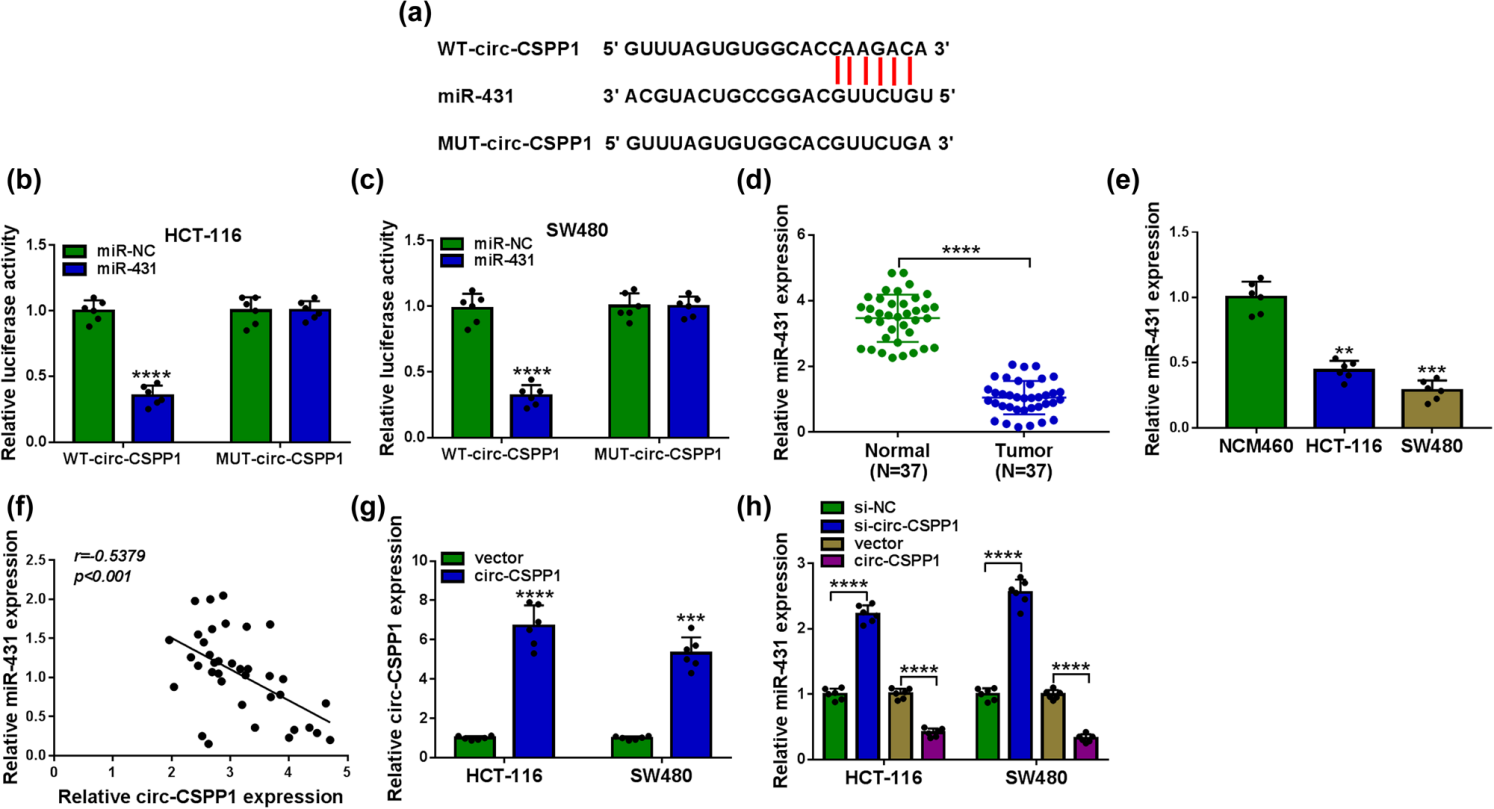
circ-CSPP1 in CRC cells directly targeted miR-431. (a) Schematic of the putative miR-431 binding region within circ-CSPP1 and the mutation of the seed region. (b and c) Dual-luciferase reporter assay in both HCT-116 and SW480 cells. (d and e) Relative miR-431 level by qRT-PCR in 37 pairs of CRC tissues and matched noncancerous tissues, NCM460, HCT-116, and SW480 cells. (f) Correlation between miR-431 and circ-CSPP1 levels in CRC tissues using Pearson’s correlation test. (g) The level of circ-CSPP1 by qRT-PCR in cells transfected with vector or circ-CSPP1 overexpression plasmid. (h) Relative miR-431 level by qRT-PCR in HCT-116 and SW480 cells transfected with si-NC, si-circ-CSPP1, vector or circ-CSPP1 overexpression plasmid. vector: negative control plasmid, circ-CSPP1: circ-CSPP1 overexpression plasmid. **P < 0.01, ***P < 0.001, or ****P < 0.0001.

To understand the mechanism by which circ-CSPP1 regulated CRC development, we used online CircInteractome database to help identify the targeted miRNAs. Of interest, a putative miR-431 binding region was predicted within circ-CSPP1 (Figure 3a). When we cloned the circ-CSPP1 segment containing the target region into a luciferase vector, the cotransfection of the luciferase reporter (WT-circ-CSPP1) and miR-431 mimic into the two CRC cells produced lower luciferase activity than cells cotransfected with mimic control, but the mutant in the seed region (MUT-circ-CSPP1) completely abolished the downregulated impact of miR-431 mimic (Figure 3b and c). Additionally, the data of qRT-PCR showed the remarkable underexpression of miR-431 in CRC tissues and cells (Figure 3d and e). Moreover, a strong inverse correlation between miR-431 and circ-CSPP1 levels was discovered in CRC tissues (Figure 3f). The transfection efficiency of circ-CSPP1 overexpression plasmid into the two cells was gauged by qRT-PCR (Figure 3g). Importantly, miR-431 expression was significantly increased by circ-CSPP1 knockdown and decreased when circ-CSPP1 is overexpressed in the two CRC cells (Figure 3h).

### 3.4 Knockdown of circ-CSPP1 regulated CRC cell progression *in vitro* by upregulating miR-431

**Figure 4:**
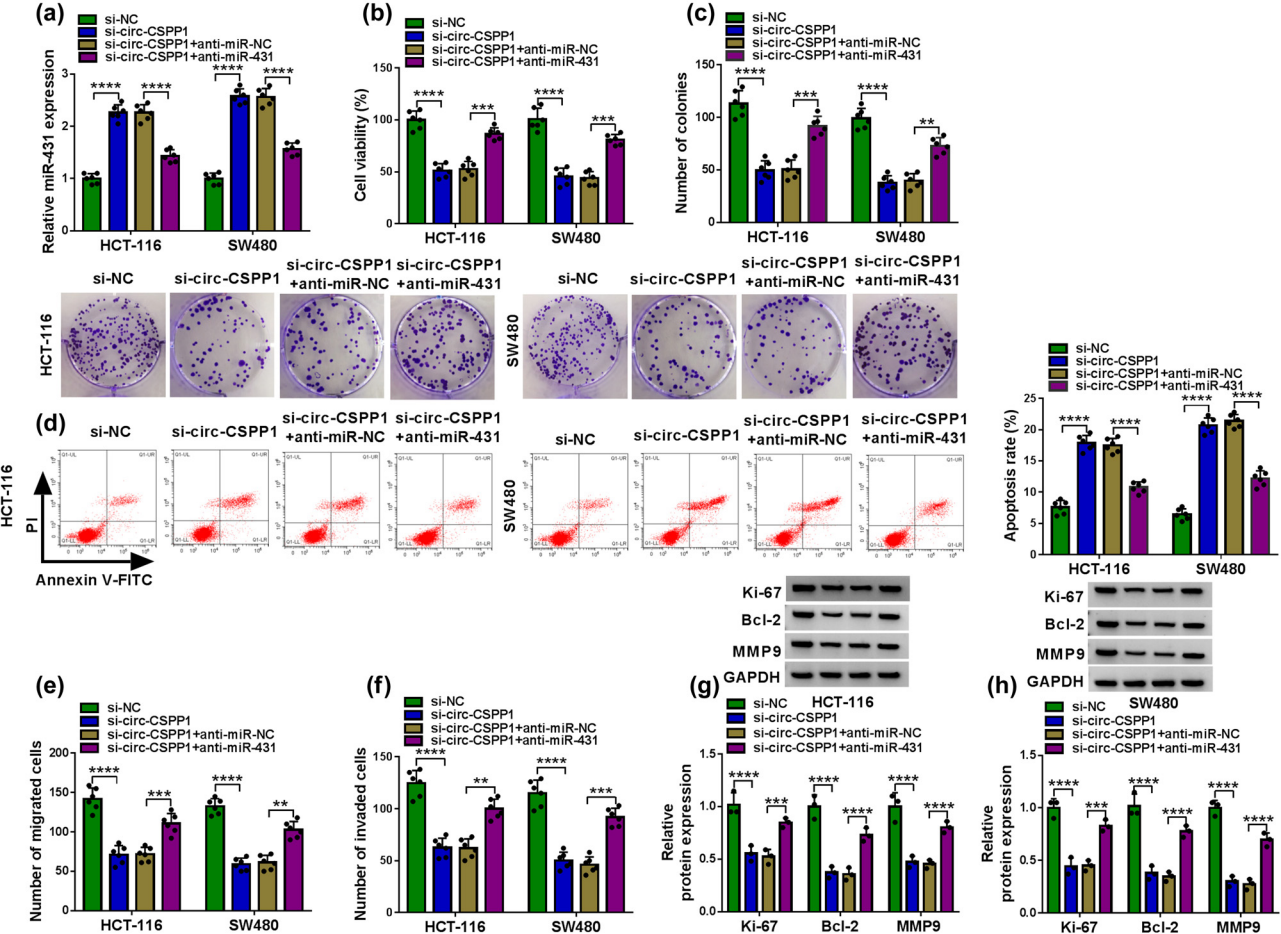
miR-431 was involved in circ-CSPP1-mediated regulation in CRC cell progression *in vitro*. HCT-116 and SW480 cells were transfected with si-NC, si-circ-CSPP1, si-circ-CSPP1 + anti-miR-NC or si-circ-CSPP1 + anti-miR-431. (a) qRT-PCR for miR-431 expression in transfected cells. (b) CCK-8 assay for cell viability. (c) Colony formation by colony formation assay. (d) Flow cytometry for cell apoptosis. (e and f) Transwell assay for cell migration and invasion. (g and h) Western blot for Ki-67, Bcl-2, and MMP9 expression levels in transfected cells. **P < 0.01, ***P < 0.001, or ****P < 0.0001.

We then determined whether miR-431 was a molecular mediator of circ-CSPP1 in regulating CRC cell progression *in vitro*. By contrast, the transfection of anti-miR-431 remarkably abrogated the augmentation of si-circ-CSPP1 on miR-431 level in both HCT-116 and SW480 cells (Figure 4a). “Rescue” experiments wherein miR-431 was underexpressed prominently abolished si-circ-CSPP1-mediated inhibition on cell viability (Figure 4b) and colony formation (Figure 4c), and promotion on cell apoptosis (Figure 4d), as well as repression on cell migration (Figure 4e) and invasion (Figure 4f). Furthermore, the reduced expression of miR-431 dramatically reversed the suppressive effect of circ-CSPP1 knockdown on Ki-67, Bcl-2, and MMP9 levels in the two cells (Figure 4g and h).

### 3.5 LASP1 in CRC cells was directly targeted and repressed by miR-431

**Figure 5:**
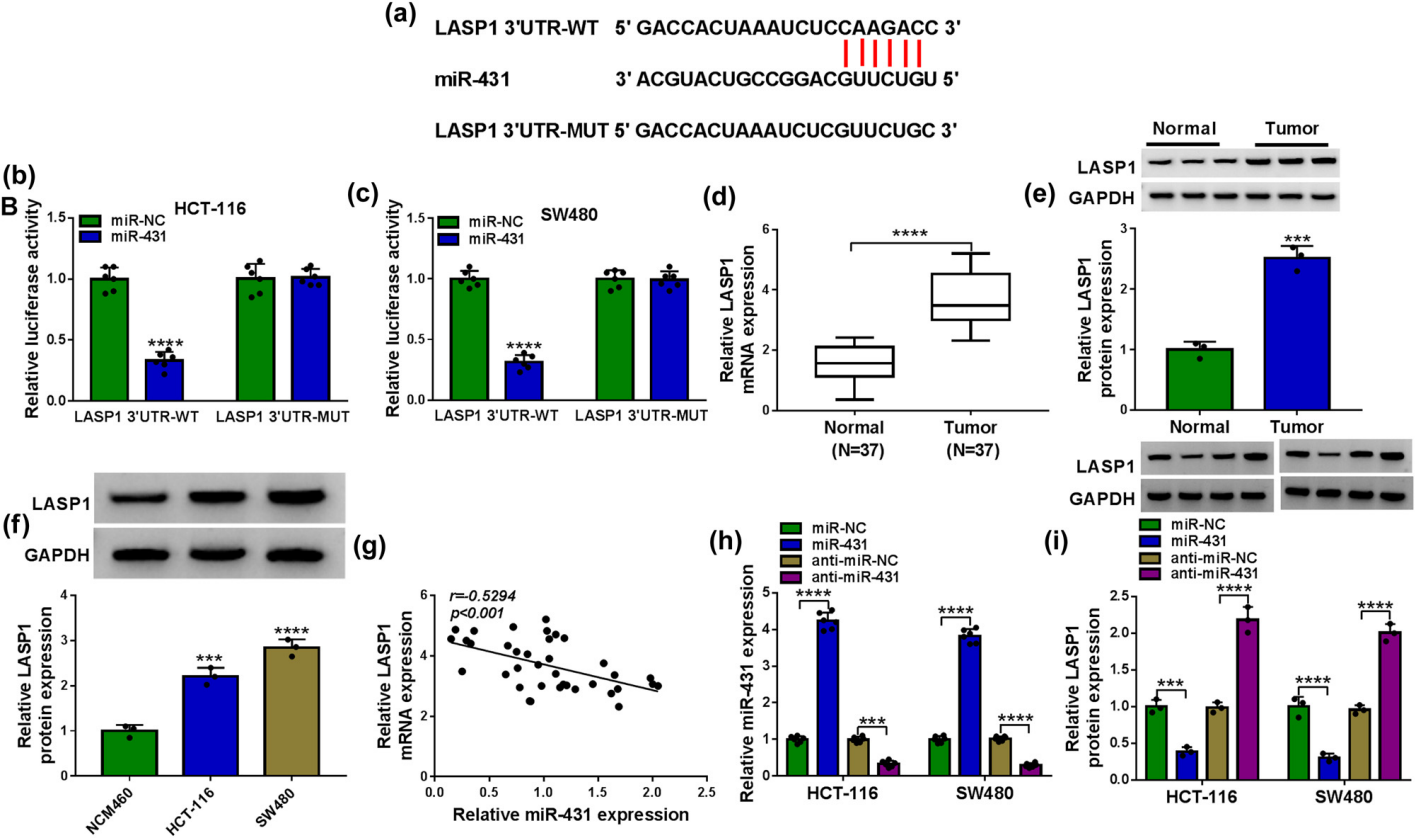
LASP1 was a direct target of miR-431 in CRC cells. (a) Schematic of the putative target sequence for miR-431 within LASP1 3′-UTR and mutated miR-431-binding sequence. (b and c) Dual-luciferase reporter assay in both HCT-116 and SW480 cells. (d–f) LASP1 mRNA expression by qRT-PCR and LASP1 protein level by western blot in CRC tissues and matched noncancerous tissues, NCM460, HCT-116, and SW480 cells. (g) Correlation between LASP1 mRNA level and miR-431 expression in CRC tissues using Pearson’s correlation test. Relative miR-431 expression by qRT-PCR (h) and LASP1 protein level by western blot (i) in HCT-116 and SW480 cells transfected with miR-NC mimic, miR-431 mimic, anti-miR-NC, or anti-miR-431. ***P < 0.001 or ****P < 0.0001.

To identify downstream effectors of miR-431, we used online target-prediction software starBase v.3 based on the presence of binding sites in the 3′-UTR. Interestingly, the predicted data revealed that LASP1 harbored a putative target sequence for miR-431 within its 3′-UTR (Figure 5a). To confirm a direct relationship between miR-431 and LASP1, we used the LASP1 3′-UTR luciferase reporters in dual-luciferase assays. The transfection of the wild-type reporter (LASP1 3′-UTR-WT) in the presence of miR-431 mimic caused a significant downregulation of relative luciferase activity, and this effect was abrogated by the mutation of the miR-431 putative target region (LASP1 3′-UTR-MUT, Figure 5b and c). The data of qRT-PCR and western blot also revealed that LASP1 was strikingly overexpressed in CRC tissues and cells (Figure 5d–f). Moreover, in CRC tissues, LASP1 mRNA level was inversely correlated with miR-431 expression (Figure 5g). We further determined whether miR-431 modulated LASP1 expression in the two CRC cells. The transfection efficiencies of miR-431 mimic or anti-miR-431 were validated by qRT-PCR (Figure 5h). As expected, LASP1 protein level was significantly reduced by the enforced expression of miR-431, while it was remarkably augmented when miR-431 depleted in both HCT-116 and SW480 cells (Figure 5i).

### 3.6 LASP1 was a functional target of miR-431 in regulating CRC cell progression *in vitro*

**Figure 6:**
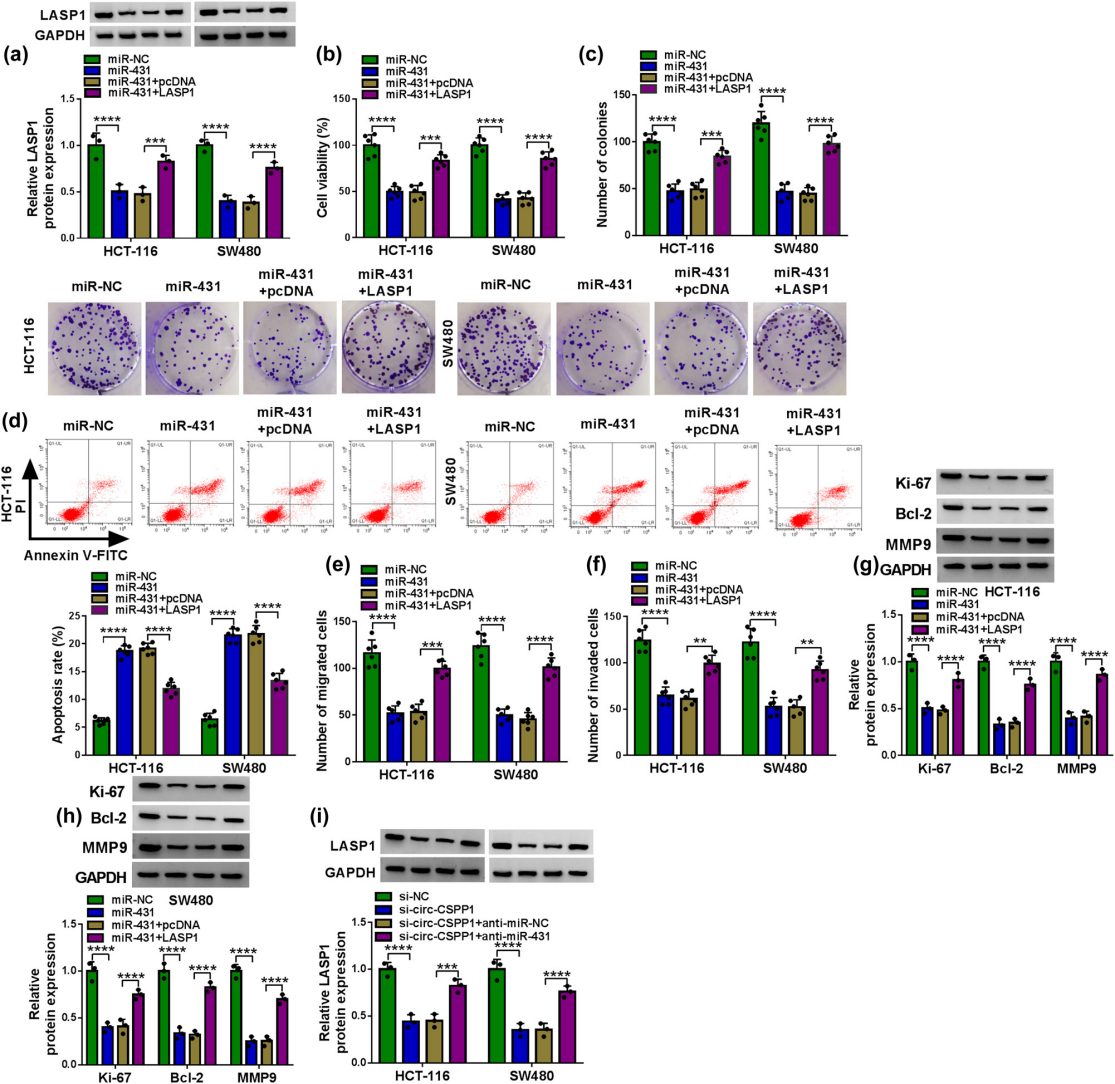
The enforced expression of miR-431 regulated CRC cell progression *in vitro* by downregulating LASP1. HCT-116 and SW480 cells were transfected with miR-NC mimic, miR-431 mimic, miR-431 mimic + pcDNA, or miR-431 mimic + LASP1, followed by the determination of LASP1 protein level by western blot (a), cell viability by CCK-8 assay (b), cell colony formation by colony formation assay (c), cell apoptosis by flow cytometry (d), cell migration and invasion by transwell assay (e and f), and Ki-67, Bcl-2, and MMP9 expression levels by western blot (g and h). (i) LASP1 protein level by western blot in HCT-116 and SW480 cells transfected with si-NC, si-circ-CSPP1, si-circ-CSPP1 + anti-miR-NC or si-circ-CSPP1 + anti-miR-431. pcDNA: negative control plasmid, LASP1: LASP1 overexpression plasmid. **P < 0.01, ***P < 0.001, or ****P < 0.0001.

In addition to the reduced effect on LASP1 expression (Figure 6a), the enforced expression of miR-431 significantly repressed cell viability (Figure 6b), colony formation (Figure 6c), and enhanced cell apoptosis (Figure 6d), as well as inhibited cell migration (Figure 6e) and invasion (Figure 6f). Moreover, miR-431 overexpression led to a striking reduction in the levels of Ki-67, Bcl-2, and MMP9 in the two CRC cells (Figure 6g and h). To provide further mechanistic insight into the link between miR-431 and LASP1 in CRC development, we elevated LASP1 level using the LASP1 overexpression plasmid in miR-431-overexpressing cells. Remarkably, the transfection of LASP1 overexpression plasmid reversed the reduction of miR-431 overexpression in LASP1 level (Figure 6a). Functional analysis results showed that the restored level of LASP1 prominently abrogated miR-431 overexpression-mediated repression on cell viability (Figure 6b) and colony formation (Figure 6c), and enhancement on cell apoptosis (Figure 6d), as well as inhibition on cell migration (Figure 6e), invasion (Figure 6f), and Ki-67, Bcl-2, and MMP9 levels (Figures 6g and h) in both HCT-116 and SW480 cells.

More interestingly, the knockdown of circ-CSPP1 induced a striking downregulation of LASP1 protein level in the two CRC cells, and this effect was dramatically abolished by anti-miR-431 (Figure 6i), demonstrating the control of circ-CSPP1 on LASP1 expression by miR-431.

### 3.7 Knockdown of circ-CSPP1 attenuated tumor growth *in vivo*

**Figure 7:**
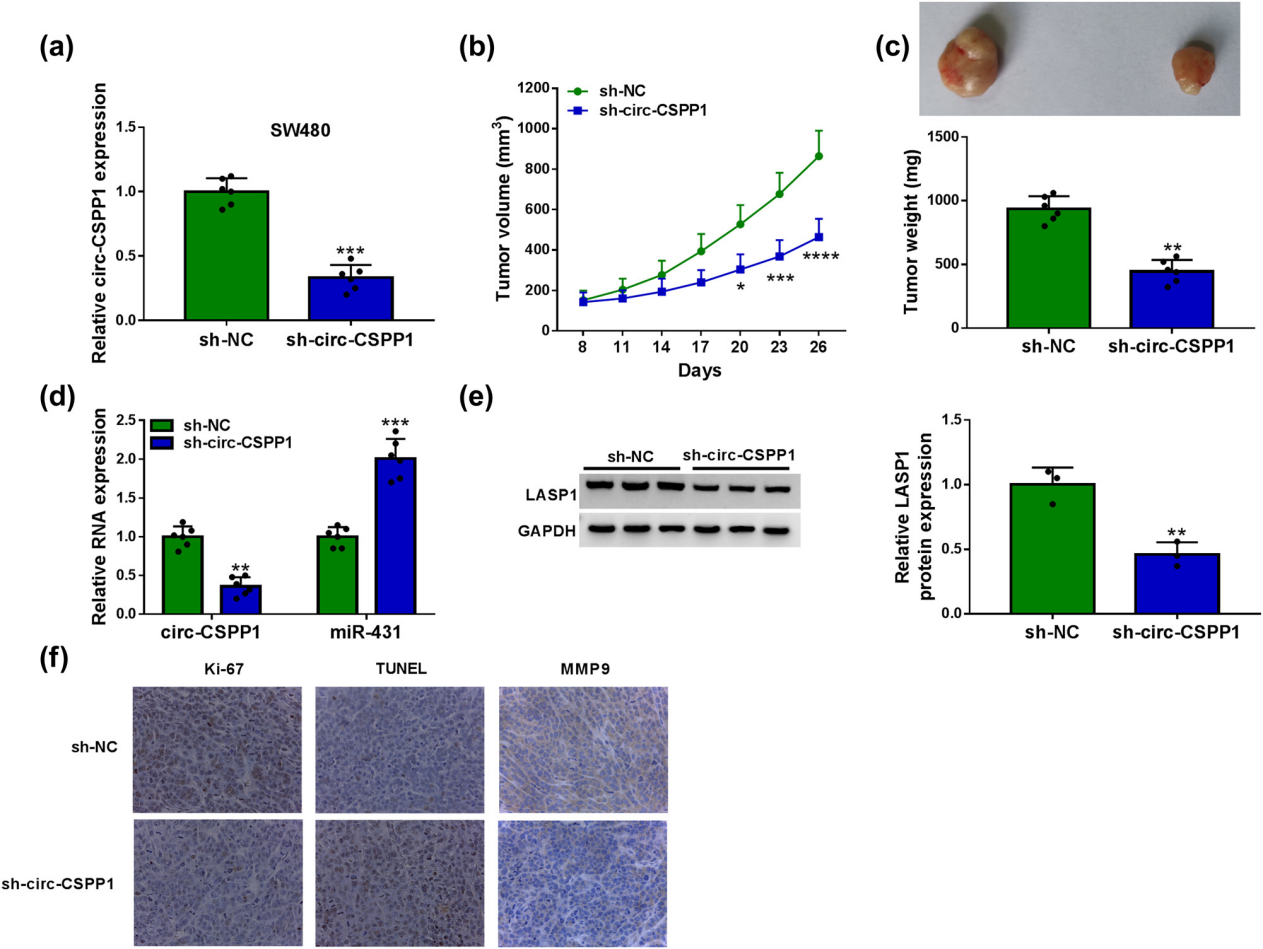
circ-CSPP1 knockdown attenuated tumor growth *in vivo*. (a) Relative circ-CSPP1 expression by qRT-PCR in sh-NC-infected or sh-circ-CSPP1-transduced SW480 cells. (b) Growth curves of the xenograft tumors formed by sh-NC-infected or sh-circ-CSPP1-transduced SW480 cells. (c) Representative images and average weight of the xenograft tumors formed by SW480 cells infected with sh-NC or sh-circ-CSPP1 at day 26 after implantation. qRT-PCR for circ-CSPP1 and miR-431 levels (d), western blot for LASP1 expression (e), and immunohistochemistry assay for Ki-67, MMP9, TUNEL (f) in the xenograft tumors derived from sh-NC-infected or sh-circ-CSPP1-transduced SW480 cells at day 26 after implantation. *P < 0.05, **P < 0.01, ***P < 0.001, or ****P < 0.0001.

We next asked whether circ-CSPP1 influenced tumor growth *in vivo*. To address this possibility, we reduced circ-CSPP1 expression in SW480 cells using a sh-circ-CSPP1 lentivirus (Figure 7a). To begin, we injected sh-NC-infected or sh-circ-CSPP1-transduced SW480 cells into the nude mice to generate the xenograft model. By contrast, the transduction of sh-circ-CSPP1 led to a significant suppression of tumor growth (Figure 7b and c). Moreover, circ-CSPP1 and LASP1 levels were remarkably downregulated and miR-431 expression was strikingly upregulated in the tumor tissues derived from the sh-circ-CSPP1-transduced SW480 cells (Figure 7d and e). Additionally, immunohistochemistry results showed that Ki-67 and MMP9 levels were decreased in sh-circ-CSPP1-transduced SW480 tumor tissues (Figure 7f), demonstrating the repression of circ-CSPP1 knockdown on tumor cell proliferation and metastasis. Furthermore, circ-CSPP1 knockdown promoted SW480 cell apoptosis confirmed by TUNEL assay (Figure 7f).

## 4 Discussion

To date, multiple lines of evidence have argued for the critical involvement of circRNAs in controlling CRC development [18,19,20]. Despite these studies, the precise actions of circRNAs in CRC development have remained unclear. circ-CSPP1 was recently identified as a circRNA whose overexpression in CRC contributed to the stemness of CRC cells [10]. Our current study expanded on this by identifying circ-CSPP1 as a crucial regulator in CRC cell malignant behaviors.

Several recent studies have reported the oncogenic role of circ-CSPP1 in ovarian cancer, cervical cancer, glioma, and hepatocellular carcinoma [21,22,23,24]. Here, our work showed a remarkable overexpression of circ-CSPP1 in CRC, in agreement with the recent work [10]. Furthermore, we presented a significant clinical association of circ-CSPP1 with the poor prognosis of CRC patients. Ki-67 is a standard marker of proliferation, and MMP9 is closely related to tumor cell metastasis [25,26]. In the current work, loss-of-function phenotypes of circ-CSPP1 uncovered that circ-CSPP1 was a positive regulator of CRC cell malignant behaviors *in vitro* and tumor growth *in vivo*. As previously reported for other circRNAs [27,28], circ-CSPP1 was stable and resistant to RNase R due to the lack of 5′ and 3′ ends [4]. Additionally, circ-CSPP1 was mainly present in the cytoplasm of CRC cells, providing the possibility for its interaction with miRNAs in the RNA-induced silencing complex where miRNAs silence gene expression [11].

Previous studies have demonstrated the conflicting roles of miR-431 in cancer development [29,30,31]. These contradictory findings might partially be due to the different tumor types in these studies, where miR-431 served as a tumor suppressor in various cancers, such as pancreatic cancer [29] and breast cancer [30], and facilitated the metastasis of pancreatic neuroendocrine tumor [31]. In the present report, we first identified miR-431 as a directly targeted miRNA of circ-CSPP1. Our data also showed the negative regulation of miR-431 on CRC cell malignant behaviors, consistent with the previous work [14]. Furthermore, our study demonstrated miR-431 as a functionally downstream mediator of circ-CSPP1 in modulating CRC cell malignant behaviors. Hu et al. reported that hsa_circ_0001742 promoted the development of tongue squamous cell carcinoma by miR-431 [32]. Li et al. highlighted that hsa_circ_0005075 functioned as a tumor promoter in hepatocellular carcinoma *via* reducing miR-431 activity [33].

LASP1, an actin-binding structural protein, is a nuclear transcriptional regulator in various cancers [34,35]. LASP1 was widely reported as a potent tumor driver in CRC via activating two survival and proliferation pathways [36,37]. In the present study, we first identified that LASP1 was a functionally important effector of miR-431 in modulating CRC cell malignant behaviors. Similarly, recent work reported that several other miRNAs, such as miR-625-5p and miR-133a, hindered CRC progression by directly targeting LASP1 [38,39]. Furthermore, our study first illuminated the modulation of circ-CSPP1 on LASP1 expression via miR-431 in CRC cells. Such analyses are hampered at present by the lack of the direct evidence *in vivo* of the novel mechanism, the circ-CSPP1/miR-431/LASP1 axis, in regulating CRC progression. Previous work reported the promoting effect of circ-CSPP1 on the stemness of CRC cells through the miR-193a-5p/COL1A1 axis [10]. Recent studies also identified the oncogenic role of circ-CSPP1 in human carcinogenesis by regulating the miR-361-5p/ITGB1 or miR-577/CCNE2 axis [22,24]. These findings suggest that there may be many other miRNA/mRNA axes that remain to be identified in the regulation of circ-CSPP1.

Collectively, the findings of the present study demonstrated the oncogenic effect of circ-CSPP1 on CRC partially by the modulation of the miR-431/LASP1 axis. We first identified the circ-CSPP1/miR-431/LASP1 axis as a novel regulatory mechanism in CRC, providing key evidence for circ-CSPP1 as a potential biomarker for CRC management.

## Appendix

**Table A1:** Sequences of qRT-PCR primers

Sequence (5′–3′)		
circ-CSPP1	Forward	CCATCCCATCAGTTCATCCT
	Reverse	TTCACCTCCAAAGAGCATCC
CSPP1 linear mRNA	Forward	CTGGAGCACAAGACCCTGAG
	Reverse	GGGAGAGGTGTCTGGAAAGC
miR-431	Forward	CGAGTGTCTTGCAGGCCGT
	Reverse	CAGTGCGTGTCGTGGAGT
U6	Forward	CTCGCTTCGGCAGCACA
	Reverse	AACGCTTCACGAATTTGCGT
GAPDH	Forward	GACAGTCAGCCGCATCTTCT
	Reverse	GCGCCCAATACGACCAAATC
LASP1	Forward	TCGGAACCATGAACCCCAAC
	Reverse	GGACTGCTTGGGGTAGTGTG

## References

[1] Bray F, Ferlay J, Soerjomataram I, Siegel RL, Torre LA, Jemal A. Global cancer statistics 2018: GLOBOCAN estimates of incidence and mortality worldwide for 36 cancers in 185 countries. CA Cancer J Clin. 2018;68:394–424. 10.3322/caac.21492 30207593

[2] Dekker E, Tanis PJ, Vleugels JLA, Kasi PM, Wallace MB. Colorectal cancer. Lancet. 2019;394:1467–80. 10.1016/S0140-6736(19)32319-0 31631858

[3] Lech G, Słotwiński R, Słodkowski M, Krasnodębski IW. Colorectal cancer tumour markers and biomarkers: Recent therapeutic advances. World J Gastroenterol. 2016;22:1745–55. 10.3748/wjg.v22.i5.1745 PMC4724606 26855534

[4] Memczak S, Jens M, Elefsinioti A, Torti F, Krueger J, Rybak A, et al. Circular RNAs are a large class of animal RNAs with regulatory potency. Nature. 2013;495:333–8. 10.1038/nature11928 23446348

[5] Xu H, Wang C, Song H, Xu Y, Ji G. RNA-Seq profiling of circular RNAs in human colorectal cancer liver metastasis and the potential biomarkers. Mol Cancer. 2019;18:8. 10.1186/s12943-018-0932-8 PMC6327571 30630466

[6] Ye DX, Wang SS, Huang Y, Chi P. A 3-circular RNA signature as a noninvasive biomarker for diagnosis of colorectal cancer. Cancer Cell Int. 2019;19:276. 10.1186/s12935-019-0995-7 PMC6829842 31700498

[7] Yuan Y, Liu W, Zhang Y, Zhang Y, Sun S. circRNA circ_0026344 as a prognostic biomarker suppresses colorectal cancer progression via microRNA-21 and microRNA-31. Biochem Biophys Res Commun. 2018;503:870–5. 10.1016/j.bbrc.2018.06.089 29928882

[8] Li XN, Wang ZJ, Ye CX, Zhao BC, Huang XX, Yang L. Circular RNA circVAPA is up-regulated and exerts oncogenic properties by sponging miR-101 in colorectal cancer. Biomed Pharmacother. 2019;112:108611. 10.1016/j.biopha.2019.108611 30797148

[9] Bian L, Zhi X, Ma L, Zhang J, Chen P, Sun S, et al. Hsa_circRNA_103809 regulated the cell proliferation and migration in colorectal cancer via miR-532-3p/FOXO4 axis. Biochem Biophys Res Commun. 2018;505:346–52. 10.1016/j.bbrc.2018.09.073 30249393

[10] Sun J, Liu J, Zhu Q, Xu F, Kang L, Shi X. Hsa_circ_0001806 acts as a ceRNA to facilitate the stemness of colorectal cancer cells by increasing COL1A1. Onco Targets Ther. 2020;13:6315–27. 10.2147/OTT.S255485 PMC7335295 32636650

[11] Bracken CP, Scott HS, Goodall GJ. A network-biology perspective of microRNA function and dysfunction in cancer. Nat Rev Genet. 2016;17:719–32. 10.1038/nrg.2016.134 27795564

[12] Verma AM, Patel M, Aslam MI, Jameson J, Pringle JH, Wurm P, et al. Circulating plasma microRNAs as a screening method for detection of colorectal adenomas. Lancet. 2015;385(Suppl 1):S100. 10.1016/S0140-6736(15)60415-9 26312830

[13] Balacescu O, Sur D, Cainap C, Visan S, Cruceriu D, Manzat-Saplacan R, et al. The impact of miRNA in colorectal cancer progression and its liver metastases. Int J Mol Sci. 2018;19:19. 10.3390/ijms19123711 PMC6321452 30469518

[14] Su WB, Liu ZY. miR-431 inhibits colorectal cancer cell invasion via repressing CUL4B. Eur Rev Med Pharmacol Sci. 2018;22:3047–52. 10.26355/eurrev_201805_15062 29863249

[15] Xu D, Wu Y, Wang X, Hu X, Qin W, Li Y, et al. Identification of functional circRNA/miRNA/mRNA regulatory network for exploring prospective therapy strategy of colorectal cancer. J Cell Biochem. 2020;121:4785–97. 10.1002/jcb.29703 32115780

[16] Ng EK, Tsang WP, Ng SS, Jin HC, Yu J, Li JJ, et al. MicroRNA-143 targets DNA methyltransferases 3A in colorectal cancer. Br J Cancer. 2009;101:699–706. 10.1038/sj.bjc.6605195 PMC2736825 19638978

[17] Chen L, Gibbons DL, Goswami S, Cortez MA, Ahn YH, Byers LA, et al. Metastasis is regulated via microRNA-200/ZEB1 axis control of tumour cell PD-L1 expression and intratumoral immunosuppression. Nat Commun. 2014;5:5241. 10.1038/ncomms6241 PMC4212319 25348003

[18] Li XN, Wang ZJ, Ye CX, Zhao BC, Li ZL, Yang Y. RNA sequencing reveals the expression profiles of circRNA and indicates that circDDX17 acts as a tumor suppressor in colorectal cancer. J Exp Clin Cancer Res. 2018;37:325. 10.1186/s13046-018-1006-x PMC6307166 30591054

[19] Li X, Wang J, Zhang C, Lin C, Zhang J, Zhang W, et al. Circular RNA circITGA7 inhibits colorectal cancer growth and metastasis by modulating the Ras pathway and upregulating transcription of its host gene ITGA7. J Pathol. 2018;246:166–79. 10.1002/path.5125 29943828

[20] Zhu M, Xu Y, Chen Y, Yan F. Circular BANP, an upregulated circular RNA that modulates cell proliferation in colorectal cancer. Biomed Pharmacother. 2017;88:138–44. 10.1016/j.biopha.2016.12.097 28103507

[21] Li QH, Liu Y, Chen S, Zong ZH, Du YP, Sheng XJ, et al. circ-CSPP1 promotes proliferation, invasion and migration of ovarian cancer cells by acting as a miR-1236-3p sponge. Biomed Pharmacother. 2019;114:108832. 10.1016/j.biopha.2019.108832 30965236

[22] Yang W, Xie T. Hsa_circ_CSPP1/miR-361-5p/ITGB1 regulates proliferation and migration of cervical cancer (CC) by modulating the PI3K-Akt signaling pathway. Reprod Sci. 2020;27:132–44. 10.1007/s43032-019-00008-5 32046405

[23] Xue YF, Li M, Li W, Lin Q, Yu BX, Zhu QB, et al. Roles of circ-CSPP1 on the proliferation and metastasis of glioma cancer. Eur Rev Med Pharmacol Sci. 2020;24:5519–25. 10.26355/eurrev_202005_21337 32495924

[24] Sun Q, Yu R, Wang C, Yao J, Zhang L. Circular RNA circ-CSPP1 regulates CCNE2 to facilitate hepatocellular carcinoma cell growth via sponging miR-577. Cancer Cell Int. 2020;20:202. 10.1186/s12935-020-01287-8 PMC7260814 32514247

[25] Juríková M, Danihel Ľ, Polák Š, Varga I. Ki67, PCNA, and MCM proteins: markers of proliferation in the diagnosis of breast cancer. Acta Histochem. 2016;118:544–52. 10.1016/j.acthis.2016.05.002 27246286

[26] Wang W, Li D, Xiang L, Lv M, Tao L, Ni T, et al. TIMP-2 inhibits metastasis and predicts prognosis of colorectal cancer via regulating MMP-9. Cell Adh Migr. 2019;13:273–84. 10.1080/19336918.2019.1639303 PMC6629184 31293204

[27] Zheng X, Chen L, Zhou Y, Wang Q, Zheng Z, Xu B, et al. A novel protein encoded by a circular RNA circPPP1R12A promotes tumor pathogenesis and metastasis of colon cancer via Hippo-YAP signaling. Mol Cancer. 2019;18:47. 10.1186/s12943-019-1010-6 PMC6440158 30925892

[28] Jia Q, Ye L, Xu S, Xiao H, Xu S, Shi Z, et al. Circular RNA 0007255 regulates the progression of breast cancer through miR-335-5p/SIX2 axis. Thorac Cancer. 2020;11:619–30. 10.1111/1759-7714.13306 PMC7049509 31962380

[29] Yang J, Zhu H, Jin Y, Song Y. miR-431 inhibits cell proliferation and induces cell apoptosis by targeting CDK14 in pancreatic cancer. Eur Rev Med Pharmacol Sci. 2018;22:4493–9. 10.26355/eurrev_201807_15503 30058687

[30] Wang W, Dong Y, Li X, Pan Y, Du J, Liu D. MicroRNA-431 serves as a tumor inhibitor in breast cancer through targeting FGF9. Oncol Lett. 2020;19:1001–7. 10.3892/ol.2019.11126 PMC6924186 31897213

[31] Zhang T, Choi S, Zhang T, Chen Z, Chi Y, Huang S, et al. miR-431 promotes metastasis of pancreatic neuroendocrine tumors by targeting DAB2 interacting protein, a Ras GTPase activating protein tumor suppressor. Am J Pathol. 2020;190:689–701. 10.1016/j.ajpath.2019.11.007 PMC7074368 31953039

[32] Hu YT, Li XX, Zeng LW. circ_0001742 promotes tongue squamous cell carcinoma progression via miR-431-5p/ATF3 axis. Eur Rev Med Pharmacol Sci. 2019;23:10300–12. 10.26355/eurrev_201912_19668 31841185

[33] Li MF, Li YH, He YH, Wang Q, Zhang Y, Li XF, et al. Emerging roles of hsa_circ_0005075 targeting miR-431 in the progress of HCC. Biomed Pharmacother. 2018;99:848–58. 10.1016/j.biopha.2018.01.150 29710484

[34] Butt E, Raman D. New frontiers for the cytoskeletal protein LASP1. Front Oncol. 2018;8:391. 10.3389/fonc.2018.00391 PMC6160563 30298118

[35] Hu Z, Wang X, Cui Y, Li C, Wang S. LASP1 in tumor and tumor microenvironment. Curr Mol Med. 2017;17:541–8. 10.2174/1566524018666180222115103 29473505

[36] Niu Y, Shao Z, Wang H, Yang J, Zhang F, Luo Y, et al. LASP1-S100A11 axis promotes colorectal cancer aggressiveness by modulating TGFβ/Smad signaling. Sci Rep. 2016;6:26112. 10.1038/srep26112 PMC4867635 27181092

[37] Zhou R, Shao Z, Liu J, Zhan W, Gao Q, Pan Z, et al. COPS5 and LASP1 synergistically interact to downregulate 14-3-3σ expression and promote colorectal cancer progression via activating PI3K/AKT pathway. Int J Cancer. 2018;142:1853–64. 10.1002/ijc.31206 29226323

[38] Shang T, Zhou X, Chen W. LINC01123 promotes progression of colorectal cancer via miR-625-5p/LASP1 axis. Cancer Biother Radiopharm. 2020. 10.1089/cbr.2020.3740 32423238

[39] Wang H, An H, Wang B, Liao Q, Li W, Jin X, et al. miR-133a represses tumour growth and metastasis in colorectal cancer by targeting LIM and SH3 protein 1 and inhibiting the MAPK pathway. Eur J Cancer. 2013;49:3924–35. 10.1016/j.ejca.2013.07.149 23968734

